# Minimally invasive repair of iatrogenic right ventricular perforation guided by bedside contrast-enhanced ultrasound: A case report and literature review

**DOI:** 10.3389/fcvm.2022.986904

**Published:** 2022-10-04

**Authors:** Yanchun Zhao, Yucheng Lin, Zhiliang Hong, Baochun Lai, Lianghua Lian, Lin Chen, Qi Xie, Xiaofen Zhou, Songsong Wu

**Affiliations:** ^1^Department of Ultrasonography, Fujian Provincial Hospital, Shengli Clinical Medical College of Fujian Medical University, Fuzhou, China; ^2^Department of Ultrasonography, Affliated Fuzhou First Hospital of Fujian Medical University, Shengli Clinical Medical College of Fujian Medical University, Fuzhou, China; ^3^Department of Cardiology, Fujian Provincial Hospital, Shengli Clinical Medical College of Fujian Medical University, Fuzhou, China

**Keywords:** cardiac perforation, leadless pacemaker, pericardial tamponade, bedside ultrasound, case report

## Abstract

This case report involves a 93-year-old female patient with atrioventricular block and suffered right ventricular free wall perforation during installation of Micra Leadless Pacemaker (MLP). Pericardial tamponade occurred shortly, and we adopted pericardial catheter drainage as the primary emergency treatment. Considering the patient's physical conditions and leveraging the special treatment facilitates of the Intensive Care Unit (ICU), we tried a new emergency treatment approach. After putting the patient under intravenous anesthesia (no cardiac arrest), we made a small intercostal incision and performed bedside minimally invasive repair of right ventricular free wall perforation. It should be noted that ultrasound played a key role in pinpointing the breach and intraoperative guidance. We first used contrast-enhanced ultrasound (CEUS) to locate the breach. Then guided by bedside ultrasound, we accessed the perforation with the minimum incision size (5 cm). Our experience in this case may serve as a good reference in the emergency treatment for right ventricular free wall perforation.

## Introduction

Implantation of permanent cardiac pacemaker is an effective treatment modality for bradyarrhythmia. MLP implantation, featuring short procedure and no pocket, has proven to be a safe and reasonable substitute for traditional transvenous pacemaker for senile patients (1). Since the MLP delivery system has a relatively large inner diameter, there is certain incidence (c. 4%) of serious adverse events. The most commonly seen and severe complication is cardiac perforation (2). Clinical manifestations vary according to the location, aperture size and the patient's blood coagulation state, and may include chest pain, dyspnea and/or tamponade. Dynamic monitoring of pericardial effusion and accurate location of the breach are perquisites for determining subsequent treatment. Transthoracic echocardiography is first choice in this regard. Most small perforations on the myocardial wall will close naturally during heart beating. Generally speaking, evidence of hemodynamic instability and pericardial tamponade are decisive criteria for emergency clinical intervention. Left ventricular perforation can rapidly lead to severe hemodynamic damage. In this scenario, emergency open surgery is the only viable salvage option. Conversely, right ventricular lesions may be stabilized without open surgery (3), and there have been reports on successful closure of right ventricular perforations with glue and devices (4, 5). Only a handful of previous reports mentioned the value of contrast-enhanced ultrasound (CEUS) in locating myocardial perforation, while no report has been made on bedside minimally invasive repair of cardiac perforation through a small intercostal incision. Our report is the first to describe a new approach for successful bedside repair of an iatrogenic right ventricular perforation with the help of CEUS.

## Case presentation

The patient is a 93-year-old woman with post-activity chest tightness and shortness of breath for 6 months. She was hospitalized after experiencing worsened symptoms for 10 days. 24-hour ambulatory electrocardiograph confirmed the diagnosis of third-degree atrioventricular block and second-degree type II atrioventricular block. The patient has a history of hypertension, hypertensive heart disease and coronary artery disease. When hospitalized, her blood pressure was 150/70 mmHg, her heart rate was 48 beats per minute, and her cardiac function was class II by NYHA criteria. No obvious abnormalities were found in her physical tests, serologic test and other biochemical tests.

**Figure 1:**
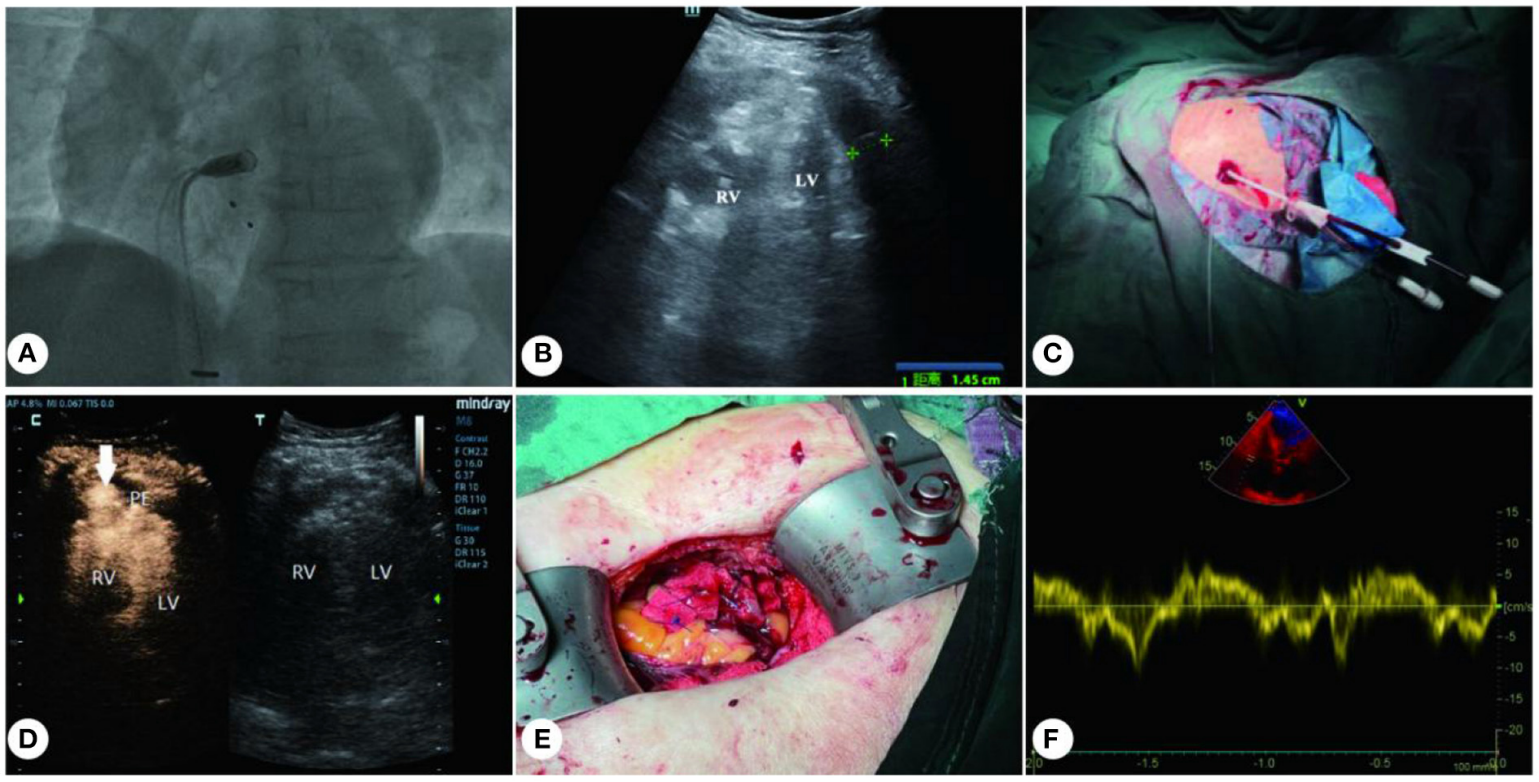
Treatment for iatrogenic right ventricular perforation. **(A)** Delivery system carrying the MLP to right ventricle. **(B)** Medium-volume effusion in the pericardial cavity. **(C)** 12F pigtail catheter placed (under ultrasound guidance) into the pericardial cavity. **(D)** CEUS shows right ventricular free wall perforation (with arrow indication). **(E)** 3-0 prolene with spacers were used to close the RV perforation. **(F)** Recovery of cardiac function after surgery. RV, right ventricle; LV, left ventricle; PE, pericardial effusion.

After clinical evaluation, we proposed implanting a MLP (Medtronic, USA) to mitigate clinical symptoms. The procedure, conducted in the interventional catheterization laboratory, was as follows: After local anesthesia, the patient's left femoral vein was punctured to place the sheath catheter, and temporary pacing electrode was placed in the apex of right ventricle. The pacing started well at a frequency of 60 beats per minute. The patient's right femoral vein was punctured and fed with a stiffened guidewire, while the transfer sheath and delivery system carrying the Micra were delivered into the middle of the right atrium successively, then the delivery system bent across the tricuspid valve to the right ventricular septum (Figure 1A). During the procedure, however, the patient became irritable and her blood pressure dropped to 98/70 mmHg. Metabolic acidosis ensued. Clinical presentations indicated high probability of cardiac perforation in the patient. Therefore, the procedure was terminated.

A multidisciplinary consultation was immediately requested. A medium dose of pericardial fluid and heart compression (Figure 1B) were identified with a Mindray M8 portable color Doppler ultrasound diagnostic instrument and a C5-1 probe (frequency: 2–5 MHz). For emergency treatment, a 12F pigtail catheter (Lingjie, Guangzhou, China) was inserted, with the guidance of ultrasound, to drain the pericardial effusion (Figure 1C). However, conventional ultrasound failed to locate the breach. After orotracheal intubation, autologous blood transfusion, blood transfusion and related medication, the patient's blood pressure temporarily recovered (145/60 mmHg), and she was immediately transferred to the ICU.

After pericardial tamponade was mitigated, the patient was injected 2 ml SonoVue (Bracco Suisse SA) with a 20 G cannula needle through the median cubital vein. Eight seconds later, microsphere contrast agent spilled into pericardial cavity through right ventricular free wall and concentrated locally (Figure 1D; Supplementary Video 1). Notably, this breach was small (inner diameter: c. 4 mm), while the outside of the perforated ventricular wall was surrounded by pericardial tissue and thrombus.

About 30 min after the breach was located, the patient once again fell into a comma, and her blood pressure dropped to 37/32 mmHg. Considering that the patient was too old to undergo open-heart surgery and there was not enough time to transfer her to the operation room, the cardiac surgeon and the ultrasound interventionalist devised a minimally invasive surgical plan for conducting bedside repair of the perforation. That is to put the patient under intravenous anesthesia (rocuronium, propofol, and remifentanil), and directly access the breach through a small intercostal incision. An ultrasonic probe was used for transverse scanning of the fourth and fifth intercostal space in the precordial region. After the breach in the right ventricular wall was located, the probe was rotated by 90 degrees to mark the breach on both the transverse and longitudinal views by pressing with a blunt instrument. The corresponding body surface position was the fourth intercostal space in the precordial region. After routine disinfection, the cardiac surgeon made 2.5 cm incisions leftward and rightward from the anchor point, and then incised the skin, subcutaneous tissue, pericardium and pleura, so as to access the breach in the right ventricular free wall. The perforation was about 6 mm in diameter, larger than that shown by CEUS, while the pericardial and myocardial tissues were brittle. Five stitches of 3-0 prolene with spacers were used to intermittently close the perforation (Figure 1E; Supplementary Video 2). When there was no more active bleeding, the pericardial blood clot was cleared and the incisions were routinely sutured. The original 12F drainage catheter was retained, and the patient's blood pressure gradually recovered during the procedure.

Two hours after the procedure, the patient's vitals recovered to HR 80 bpm, R 26 bpm, BP 88/37 mmHg, SPO2 100%, and she was successfully resuscitated. Two days after the surgery, transthoracic echocardiography results showed that the patient's cardiac function was restored, with left ventricular ejection fraction at 50% (Figure 1F). After being observed for 10 days to ensure hemodynamic stability, the patient underwent permanent transvenous pacemaker implantation (Medtronic, Minneapolis, Minnesota). Two weeks after the implantation, the patient recovered well and was discharged from the hospital.

## Discussion

**Figure 2:**
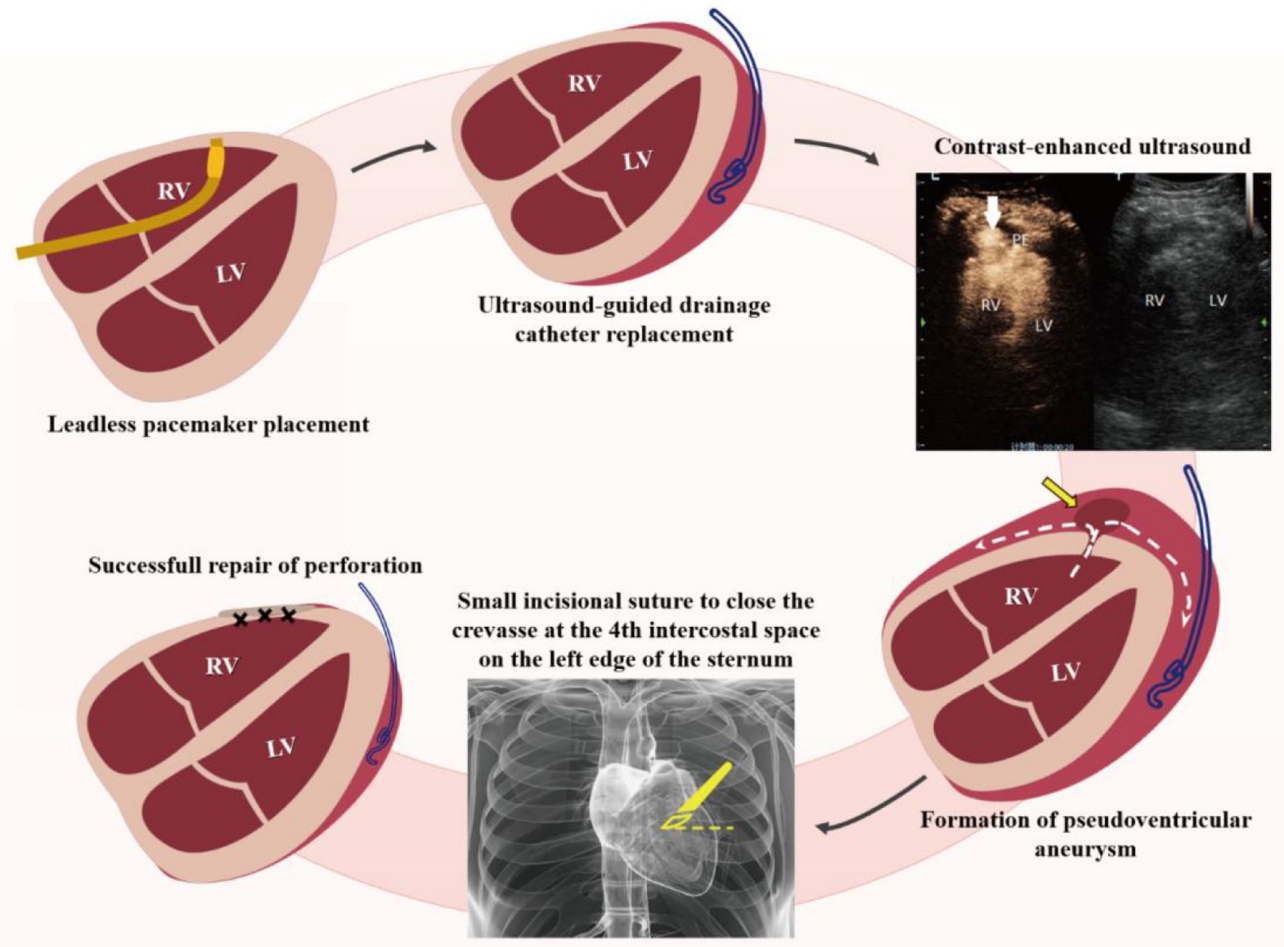
Schematic diagram of treatment for iatrogenic right ventricular perforation. RV, right ventricle; LV, left ventricle; PE, pericardial effusion.

This case report provides a new emergency treatment—minimally invasive repair of right ventricular free wall perforation with the help of bedside ultrasound. While treating a senile patient with an iatrogenic right anterior ventricular wall perforation, we used CEUS to pinpoint the breach and performed a successful ultrasound-guided bedside repair through a small intercostal incision (Figure 2).

The probability of cardiac perforation or pericardial tamponade during MLP implantation is 1.6% (1). Giudici et al. (6) have successfully used the strategy of temporary-permanent pacemaker in MLP implantation, which is applicable to severely ill patients with underlying diseases and poor prognoses, and can reduce complications. Our placement plan also followed this strategy, but cardiac perforation occurred anyway. We suspect that the perforation resulted from the sheath catheter touching the fragile myocardium during the delivery of the Micra. Research findings show that female, low BMI, history of myocardial infarction and/or pulmonary diseases are significant factors that may cause cardiac perforation during MLP implantation (7). The patient herein is a senile woman with several underlying diseases; her conditions are a key factor causing cardiac perforation.

Bedside ultrasound plays a key role in early detection of pericardial effusion and pericardial tamponade. However, conventional ultrasound often fails to pinpoint the breach since the perforation is of a small inner diameter. Our case proved that CEUS has additional diagnostic value in this regard. Mittle et al. (8) also reported that CEUS can better diagnose cardiac rupture. Immediate intervention should be performed if spillage of contrast agent into the pericardial cavity or evidence of pseudoaneurysm is detected. Other cases also proved that focused transesophageal echocardiography (TEE) is a safe and efficient means for diagnosing an iatrogenic cardiac perforation in the posterior lateral free wall (9). It should be emphasized that not all cases of myocardial rupture will present spillage of ventricular contrast agent into the pericardial space, as some cases may present a local filling defect due to obstruction of the rupture site by thrombus (8).

Bedside pericardiocentesis is an essential procedure for emergency care, while ultrasound can play a key role in preventing myocardial re-injury. For right ventricular perforation, interventional procedure can be implemented to close the breach when the patient is hemodynamically stable (10). Such a procedure requires that the catheterization laboratory is equipped with corresponding hardware and expertise (4, 5). Thoracotomy for repair of cardiac perforation in the presence of life-threatening complications is an effective therapy (3). However, such a procedure is not applicable to some patients, due to the high risks of thoracotomy, advanced age and underlying diseases. Take our case for example. Sudden recurrence of pericardial tamponade left no time for transferring the patient to the operating room or the interventional catheterization laboratory. In this context, emergency bedside surgery was a new and daring attempt.

To the best of our knowledge, we reported the first case of a senile patient undergoing successful CEUS-guided bedside minimally invasive repair of an iatrogenic ventricular wall perforation through a small intercostal incision. With no need for sternotomy and temporary cardiac arrest, the patient was only under intravenous anesthesia. Guided by bedside ultrasound, we accessed the breach through a transverse incision along the intercostal space. Such a procedure, featuring smaller surgical trauma, can serve as an alternative of bedside emergency treatment. The three key factors of the successful procedure are: (1) CEUS can help pinpoint the perforation and evaluate its severity; (2) The breach was close to the thoracic wall, allowing ultrasound-guided direct access through a small intercostal incision; and (3) The breach was small, and the intraoperative blood extravasation was slow as a result of weakened heart beating, which made bedside procedure viable. Our experience in this case may serve as a good reference in the emergency treatment for right ventricular free wall perforation.

## Conclusion

To sum up, bedside ultrasound plays a key role in the diagnosis of and treatment for cardiac perforation, while CEUS can enhance diagnosis of small perforations on the myocardial wall. For right ventricular free wall perforation in life-threatening emergencies, bedside ultrasound-guided small-incision surgical repair is an alternative emergency treatment modality, which helps avoid unnecessary cardiac surgery with sternotomy.

## Data availability statement

The original contributions presented in the study are included in the article/Supplementary material, further inquiries can be directed to the corresponding author/s.

## Ethical statement

This study, involving a human participant, has been reviewed and approved by Fujian Provincial Hospital. The participant has provided her written informed consent to participate in this study. Written informed consent was obtained from the individual for the publication of any and all images or data included in this report.

## Author contributions

YZ followed the patients and wrote the paper. SW, BL, LL, LC, QX, and XZ participated in the treatment of patients. YL contributed in picture editor. All authors contributed to the article and approved the submitted version.

## Funding

This study was funded by a grant (grant number: 2020J011090) from the Natural Science Foundation of Fujian Province.

## Conflict of interest

The authors declare that the research was conducted in the absence of any commercial or financial relationships that could be construed as a potential conflict of interest.

## Publisher's note

All claims expressed in this article are solely those of the authors and do not necessarily represent those of their affiliated organizations, or those of the publisher, the editors and the reviewers. Any product that may be evaluated in this article, or claim that may be made by its manufacturer, is not guaranteed or endorsed by the publisher.

## Supplementary material

Supplementary Video 1 Contrast-enhanced ultrasound (CEUS) of ventricular wall perforation.

Supplementary Video 2 Minimally invasive repair of cardiac perforation.

## Abbreviations

MLP: Micra Leadless Pacemaker
CEUS: contrast-enhanced ultrasound.
